# A New Cypovirus-1 Strain as a Promising Agent for Lepidopteran Pest Control

**DOI:** 10.1128/spectrum.03855-22

**Published:** 2023-05-08

**Authors:** Vyacheslav V. Martemyanov, Yuriy B. Akhanaev, Irina A. Belousova, Sergey V. Pavlusin, Maria E. Yakimova, Daria D. Kharlamova, Alexander A. Ageev, Anna N. Golovina, Sergey A. Astapenko, Alexey V. Kolosov, Grigory G. Ananko, Oleg S. Taranov, Alexander N. Shvalov, Sergey A. Bodnev, Nikita I. Ershov, Inna V. Grushevaya, Maxim A. Timofeyev, Yuri S. Tokarev

**Affiliations:** a Laboratory of Ecological Physiology, Institute of Systematics and Ecology of Animals SB RAS, Novosibirsk, Russia; b Institute of Biology, Irkutsk State University, Irkutsk, Russia; c Biological Institute, National Research Tomsk State University, Tomsk, Russia; d Department of Information Biology, Novosibirsk State University, Novosibirsk, Russia; e Center of Forest Pyrology, Development of Forest Ecosystem Conservation, Forest Protection and Regeneration Technologies, branch of All-Russia Research Institute of Silviculture and Mechanization of Forestry, Krasnoyarsk, Russia; f FBRI State Research Center of Virology and Biotechnology VECTOR, Koltsovo, Novosibirsk Region, Russia; g Institute of Cytology and Genetics SB RAS, Novosibirsk, Russia; h All-Russian Institute of Plant Protection, Pushkin – St. Petersburg, Russia; State Key Laboratory of Microbial Resources, Institute of Microbiology, Chinese Academy of Sciences

**Keywords:** Lepidoptera, Lymantria dispar, Reoviridae, baculovirus, bioinsecticides, cypovirus, host range, insect, optical brightener, pest control

## Abstract

Now more than ever researchers provide more and more evidence that it is necessary to develop an ecologically friendly approach to pest control. This is reflected in a sharp increase in the value of the biological insecticide market in recent decades. In our study, we found a virus strain belonging to the genus *Cypovirus* (Reoviridae); the strain was isolated from *Dendrolimus sibiricus*, possessing attractive features as a candidate for mass production of biological agents for lepidopteran-pest control. We describe the morphological, molecular, and ecological features of the new *Cypovirus* strain. This strain was found to be highly virulent to *D. sibiricus* (the half-lethal dose is 25 occlusion bodies per second-instar larva) and to have a relatively wide host range (infecting representatives of five families of Lepidoptera: Erebidae, Sphingidae, Pieridae, Noctuidae, and Lasiocampidae). The virus strain showed a strong interaction with a nontoxic adjuvant (optical brightener), which decreased the lethal dose for both main and alternative hosts, decreased lethal time, and may expand the host range. Moreover, we demonstrated that the insecticidal features were preserved after passaging through the most economically suitable host. By providing strong arguments for the possible use of this strain in pest control, we call on virologists, pest control specialists, and molecular biologists to give more attention to the *Cypovirus* genus, which may lead to new insights in the field of pest control research and may provide significant advantages to compare with baculoviruses and Bacillus thuringiensis products which are nowadays main source of bioinsecticides.

**IMPORTANCE** In this article, we describe a newly discovered cypovirus strain that displays features ideally suited for the development of a modern biological insecticide: high potency, relatively broad host range, true regulating effect, flexible production (possibility to choose host species for production), interaction with enhancing adjuvants, and ecologically friendly. Based on an alignment of CPV genomes, we suggest that the enhanced host range of this new strain is the sequence of evolutionary events that occurred after coinfections involving different CPV species within the same host. These findings suggest that we need to positively reconsider CPVs as prospective agents as biocontrol products.

## INTRODUCTION

The large increases in the scale of agriculture and forestry in the last century—which are necessary for the functioning of human populations at their current density—have fostered the emergence of new pest species. The simplest way to control the population size of pests is the use of chemical insecticides. However, chemical insecticides are not ecologically friendly products. An alternative way to manage the density of pests is to use biological agents, such as natural enemies or diseases. We see a substantial rise in the bioinsecticide market, from a modest size in the 1990s to a multibillion-dollar business today that is growing at a much faster rate than the conventional crop protection industry (https://www.bpia.org/markets-for-biological-products-global-market-landscape/). The market size of agricultural biologicals was estimated to be $8.8 billion in 2019 and is projected to grow at a CAGR of 13.6% and reach $18.9 billion by 2025 (Agricultural Biologicals Market report [https://www.fortunebusinessinsights.com/industry-reports/agricultural-biologicals-market-100411]). Detailed analysis of the structure of this market reveals unequivocal dominance of Bacillus thuringiensis (Bt) based on the offered products (1). The key reasons for this state of affairs are the possibility of cultivation of this bacterium on a commercial medium and the relatively broad host spectrum of this bioinsecticide, which allows users to apply the same bioinsecticide against different representatives of one order (e.g., against Lepidoptera, Diptera, or Coleoptera). However, a serious drawback of Bt-based products is the absence of a true pest-population-regulating effect, which increases application frequency against polyvoltine pest species in a given season (2). Viral bioinsecticides based on entomopathogenic viruses (mostly representatives of genera *Nucleopolyhedrovirus* [NPV] and *Granulovirus*; Baculoviridae) are free from this disadvantage owing to vertical transmission via survival of some hosts after infection (3). In combination with high specificity to a target host, this feature has allowed these products to secure a good percentage of the market (1). The basic limitations of virus-based products are the current impossibility of mass cultivation in cultured cells (because of the two-step ontogenesis of such viruses and high prices of cell culture media [1]) and very high host specificity. This situation necessitates breeding a specific host species for each viral product (i.e., a lack of universality of a given product in a shifting market).

In this article, we describe a new strain of an RNA virus from the *Cypovirus* (CPV) genus (Reoviridae family), which has been shown to possess important features in terms of its effectiveness, productivity, host range, production prospects, interaction with an adjuvant, and overall good potential for the organic-food industry. CPVs have segmented double-stranded RNA genomes (usually 10 segments) that are about 20 to 30 kbp in size. Cypovirus virions are single-shelled capsids and have icosahedral shapes (4). CPVs, also known as cytoplasmic polyhedrosis viruses, usually cause chronic infection in the gut of insects (5, 6) and often participate in coinfection with baculoviruses (7, 8). When ingested by larvae, CPV polyhedra are dissolved in the midgut by alkaline insect juices. Then, released occlusion-derived virions infect the cells lining the gut (9, 10). The virion core is transcriptionally active and produces capped mRNAs (10). Virus infection in larvae is generally restricted to the columnar epithelial cells of the midgut, although virus replication was also reported in the fat body (11). High amounts of viral particles are also detected in the larval hemolymph (9). A distinctive feature of CPV relative to other members of the *Reovirdae* family is the incorporation of the virus particles into large (several microns) structures, polyhedra, during the late stages of infection (10). Filled with polyhedra, the gut is enlarged and acquires a milky-white color. In the case of aggressive strains, dysfunction of the larval midgut leads to the death of individuals (9). Feces of sick larvae are contaminated with viral polyhedra, thus contributing to the horizontal transmission of CPVs (9, 11).

The ability of CPVs to often form chronic infections has attracted much less attention within the scientific community than typical “killer” pathogens such as the *Baculoviridae* family (16,000 articles for search term “Baculovirus” versus 200 articles for search term “Cypovirus”). Such intense research attention has given rise to a new scientific field related to baculoviruses: baculovirus-based *in vitro* expression systems, which enable investigators to produce target proteins in appreciable amounts (12). The balance of research attention to these two groups of occlusion body-forming viruses is dramatically skewed toward the Baculoviridae if we compare the pest management products developed from these viruses: there are many nucleopolyhedrovirus-based and granulovirus-based bioinsecticides (13, 14) versus a few bioinsecticides based on *Dendrolimus punctatus* CPV (14). In spite of the described imbalance in the knowledge between Baculoviridae and *Cypovirus*, it is known that CPVs infect mostly lepidopteran pests (6, 9, 10), with rare cases of dipteran infections reported (15). No cross-order infection of CPV has been found (9), although some degree of cross-family infection has been reported in previous studies (16). Because most CPVs examined to date do not have marked pathological effects in pest hosts, interest in estimating their host range has been limited, with such studies having been carried out only sporadically and involving only a few alternative hosts from the same order (16) or involving only within-family comparisons (15).

The main aim of the present study was to describe the morphological, molecular, and biological features of a new, highly virulent strain of CPV isolated for the first time from *Dendrolimus sibiricus* (Lepidoptera, Lasiocampidae) (DsCPV-1), which is a major forest pest in boreal forests of Asia. *D. sibiricus* is an economically important pest that has attracted much attention, not only from scientists where this pest occurs naturally (17, 18), but also from scientists working in jurisdictions where this pest poses a very serious invasion risk (19, 20). Thus, it is very important to develop ecologically friendly approaches to control this pest species. Another aim of our study was to assess the host range of this virus strain, including a species that is already adapted to artificial, inexpensive mass rearing. Finally, we were interested in assessing the possible synergistic effect of a nontoxic adjuvant such as an optical brightener as these compounds have shown strong synergistic effects on target mortality when used with NPVs (21). Such data will facilitate the development of a virus-based pest control product for operational field applications.

## RESULTS

### Symptoms of the infection in the larvae, and the description of DsCPV-1.

**FIG 1:**
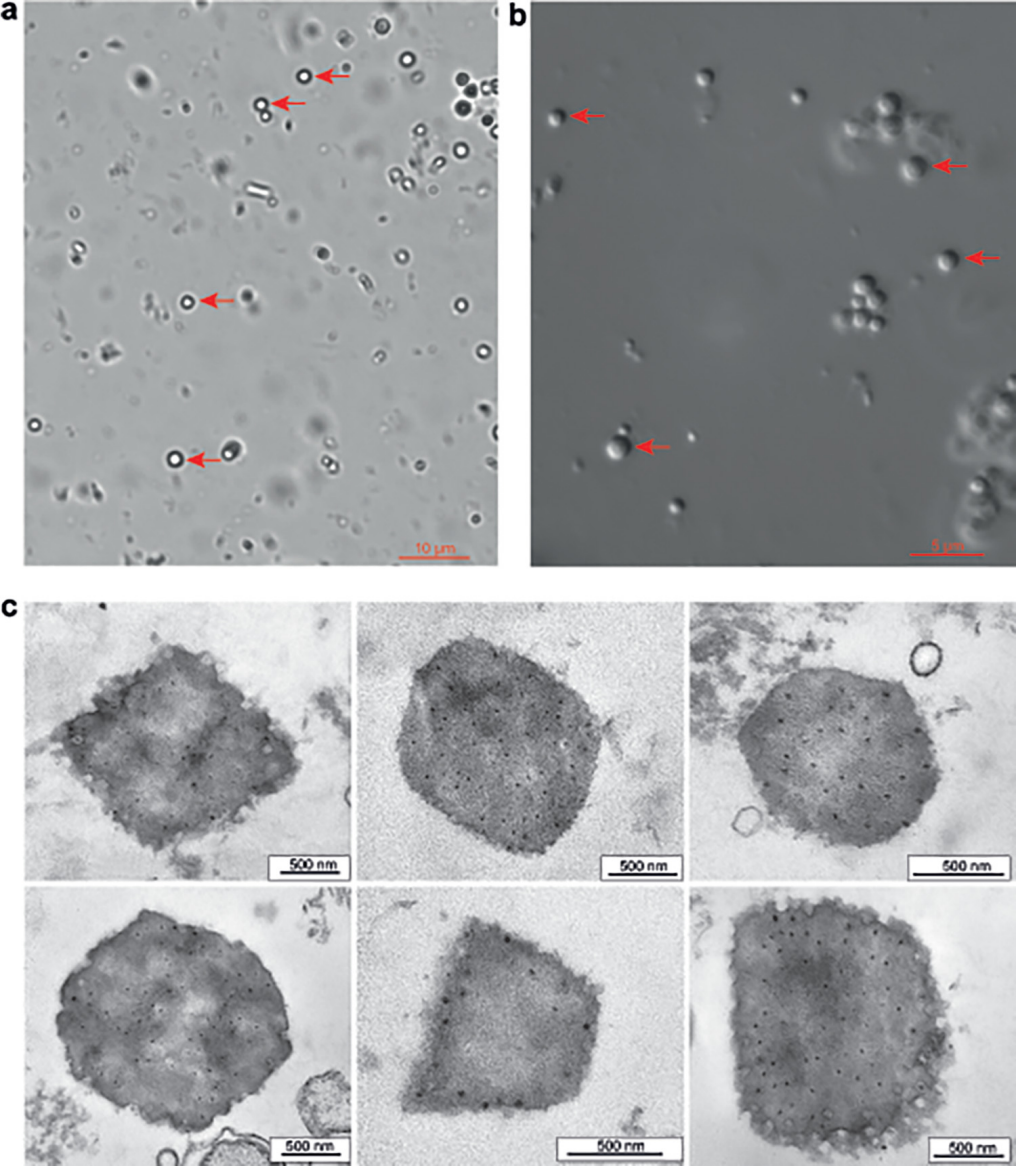
Micrographs of DsCPV-1: (a) light microscopy (arrows indicate polyhedra); (b) light microscopy with Nomarski contrast (arrows indicate polyhedra); (c) transmission electron microscopy of polyhedra of different shapes.

**FIG 2:**
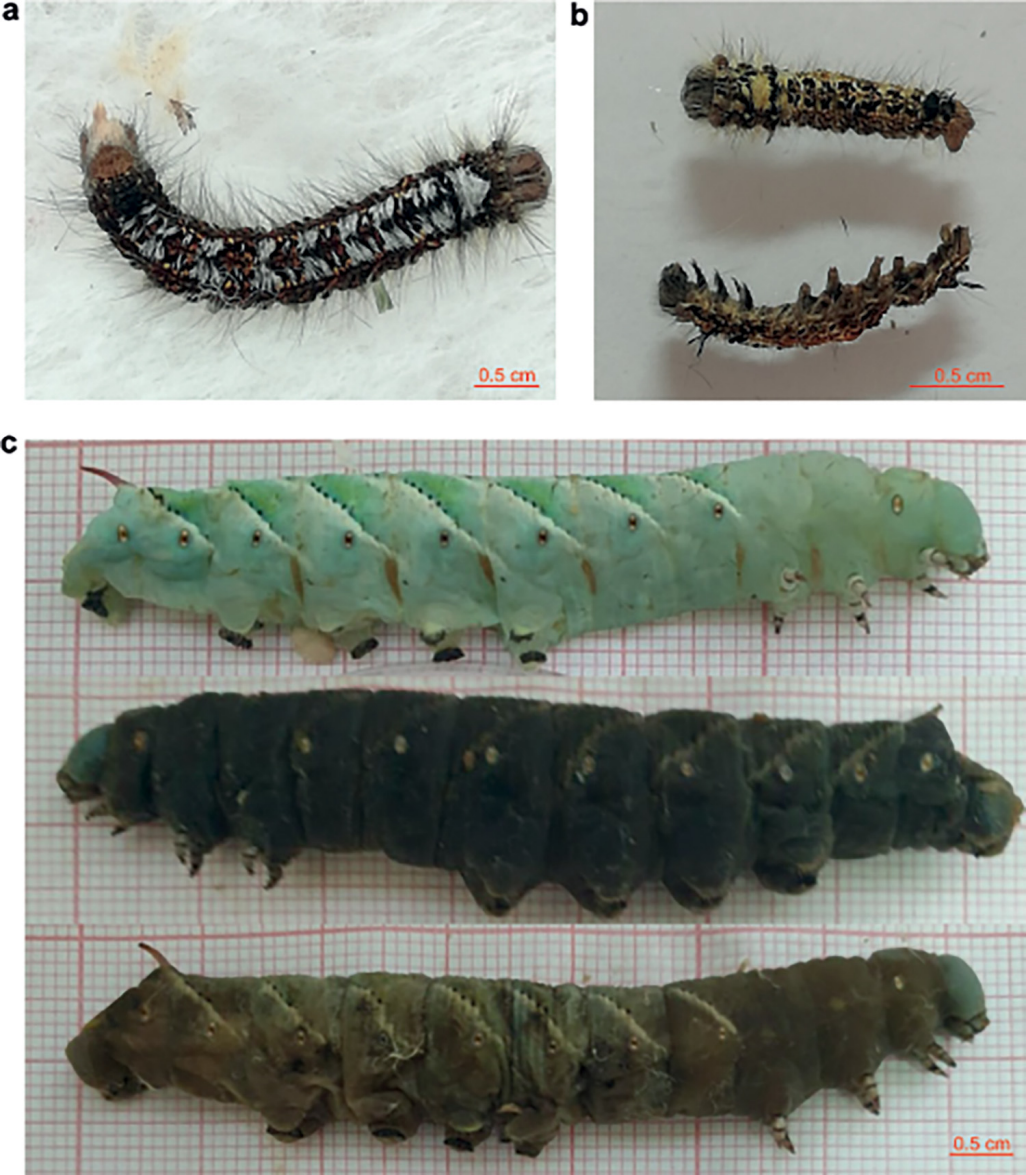
Larvae killed by DsCPV-1. (a) Late-instar *D. sibiricus* larva; (b) middle-instar *D. sibiricus* larvae killed by the bioassay; (c) dead late-instar *M. sexta* larvae.

**FIG 3:**
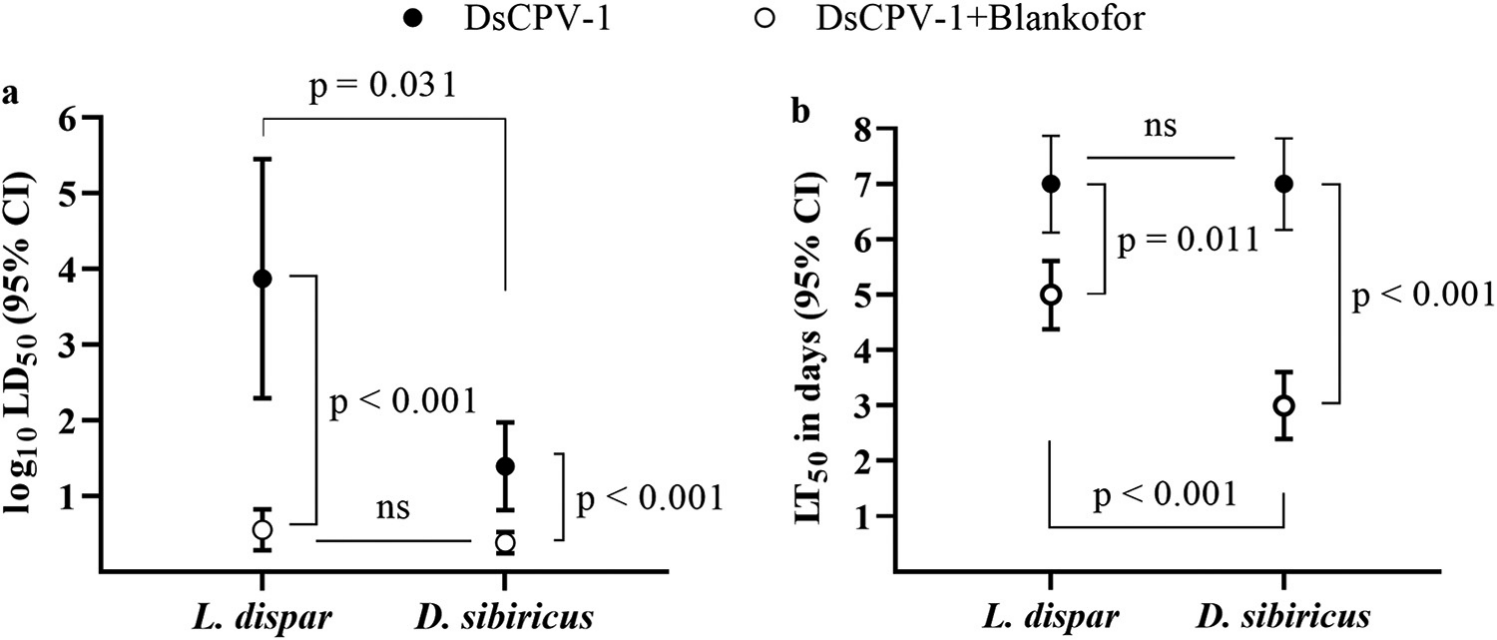
The virulence of DsCPV-1 toward second-instar larvae of *D. sibiricus* and *L. dispar*. (a) LD_50_ of DsCPV-1 combined with the optical brightener (Blankophor); (b) LT_50_ of DsCPV-1 combined with Blankophor.

The dead larvae of *D. sibiricus* were collected in an outbreak part of a pest’s range in the taiga zone (near the Eastern Sayan mountain; 55.10° N 96.58° E) in 2020. Light microscopic analysis of the dead larvae revealed many polyhedral occlusion bodies with a size of 1.5 to 2.0 μm (Fig. 1a and b). The dead larvae did not become liquefied, indicating the absence of NPV (Fig. 2a). Transmission electron microscopy confirmed the presence of spherical virus particles (with an average size of 50 to 60 nm) occluded by a crystal protein that is typical for CPV. The polyhedra had different shapes, including icosahedral (Fig. 1c). The infection of the main host (second-instar larvae of *D. sibiricus*) showed LD_50_ of 25 polyhedra per larva at 12 days (Fig. 3a). It should be noted that even at the lowest dose of DsCPV-1 (0.5 polyhedra per larva), we registered a larval mortality rate of 53% throughout the whole larval stage (Fig. S1). Mature polyhedra were also detectable at a high concentration in the feces of the infected larvae and larvae were not liquefied again (Fig. 2b). Median productivity of DsCPV-1 during the infection of middle-instar larvae was 10^8^ polyhedra/larvae with a range of 2.5 × 10^7^ to 3.1 × 10^8^.

Just as in other known type 1 CPVs, the sequenced double-stranded-RNA genome of DsCPV-1 is composed of 10 linear segments with a total length of 24.7 kb (Table S1). The genome encodes 10 proteins, including four structural capsid proteins (capsid shell protein/VP1, A-spike protein/VP2, turret protein/VP3, and large protrusion protein/VP5), two constituents of transcription enzyme complex (RNA-dependent RNA Polymerase and NTPase/VP4), and four putative nonstructural proteins (p101/NSP5, p44/NSP8, NS5/NSP9, and polyhedrin). Phylogenetic trees obtained independently for each of the proteins from a wide range of CPVs assigned the virus to the CPV 1 group (Fig. S2). Nevertheless, substantial variation in the topology of some CPV groups across the inferred trees was observed.

**FIG 4:**
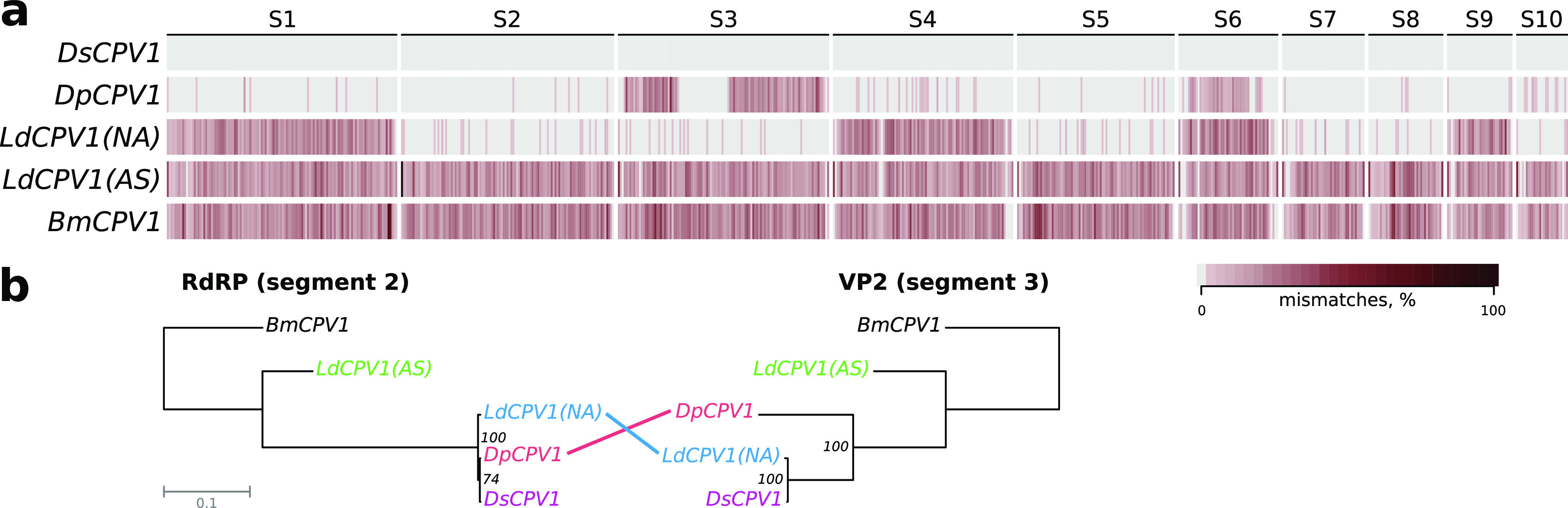
(a) A heatmap of genome-wide sequence differences between *D. sibiricus* CPV1 (DsCPV-1) and available complete genomes of other type 1 CPVs. Color intensity corresponds to the proportion of mismatches per genomic window, as defined in the color key. (b) Topological incongruence between the Maximum likelihood estimation trees of segments 2 and 3 encoding RNA-dependent RNA polymerase (RdRp) and structural protein VP3, respectively. The numbers near the branches are Felsenstein’s bootstrap support.

DsCPV-1 seems to be the closest relative of *D. punctatus* CPV-1 (DpCPV1), because all their proteins, except A-spike protein, share more than 99% identity (Table S1). Interestingly, A-spike protein, which is implicated in receptor binding and membrane penetration (22), is most similar to that of *L. dispar* CPV-1 isolate, LdCPV-1(NA). The same mosaicism is clearly observable in multiple sequence alignment of five available complete CPV-1 genomes: e.g., the third segment of DsCPV-1 is much closer to LdCPV-1 than to DpCPV-1 (Fig. 4a). Together with the corresponding incongruence between phylogenies of RdRP and A-spike protein (Fig. 4b; Fig. S2), this finding indicates that some recombination events have occurred (horizontal transmission) between different CPV strains. Accordingly, the 3SEQ analysis of mosaicism predicted that the third segment of DsCPV-1 is most likely a result of recombination between DpCPV1 and LdCPV1 (*P* = 3e-276).

### Examination of the CPV host range and the effect of the optical brightener on CPV potency.

The qualitative bioassay of other potential lepidopteran hosts revealed that *P. brassicae*, *M. brassicae*, *H. cunea*, *L. dispar*, and *M. sexta* are susceptible to DsCPV-1 infection, whereas *O. nubilalis*, G. mellonella, *L. sticticalis*, and *H. armigera* are not affected by the virus challenge (Table 1). We choose *M. sexta* as the most promising producer of DsCPV-1 based on the speed of insect development/virus productivity/costs for rearing. It is important to note that there is variation in phenotypical symptoms of dead larvae infected by DsCPV-1: some larvae become dark while others keep their initial color (Fig. 2c). After DsCPV-1 was passaged through *M. sexta* larvae, reisolated, and fed to *D. sibiricus* larvae, this virus preserved its pathogenicity toward the main host: infection by 10^4^ polyhedra per second-instar *D. sibiricus* larva killed all the tested larvae. We also compared this virulence of DsCPV-1 with its virulence toward another alternative host: *L. dispar*. We demonstrated that the susceptibility of *L. dispar* to DsCPV-1 was significantly lower than that of the main host (Fig. 3a and b).

**TABLE 1:** The Bioassay of *Ds*CPV 1 and condition of challenging during host range study

Species name (family)	Larvae instar	Used concn, polyhedra/mL	Infection method	Volume of suspension per larvae, μl^*a*^	Type of infection (individual vs group)	Total no. of larvae per treatment	no. of replicates	Duration of observation, days	Total mortality, % ±SE	Statistics
Lymantria dispar L. (Eribidae)	2	0	drop-feeding	0,5	Individual	38	4	15	0.00	χ^2^ = 61.36, df = 1, *p* < 0.001
		10^3^				48	5		8.00 ± 3.74	
		10^4^				40	4		10.00 ± 7.07	
		10^5^				40	4		17.50 ± 11.80	
		10^6^				43	5		35.66 ± 4.71	
		10^7^				41	4		72.50 ± 11.08	
Pieris brassicae L. (Pieridae)	3	0	drop-feeding	1	Individual	40	2	14	37.50 ± 2.50	χ^2^ = 32.77, df = 1, *p* < 0.001
		10^6^				45	2		92.5 ± 7.50	
Ostrinia nubilalis Hubner (Crambidae)	3	0	diet incorporation	1,000*	Group	50	1	10	20.00	χ^2^ = 0.19, df = 1, *p* = 0.660
		10^6^			Group	54	1		16.70	
Hyphantria cunea Drury (Noctuidae)	3	0	diet incorporation	1,000*	Group	48	4	15	2.08 ± 2.08	χ^2^ = 8.34, df = 1, *p* < 0.05
		10^6^			Group	46	4		4.17 ± 2.41	
		10^7^			Group	44	4		23.07 ± 6.51	
Mamestra brassicae L. (Noctuidae)	2	0	diet incorporation	1,000*	Group	54	3	14	42.59 ± 7.40	χ^2^ = 28.93, df = 1, *p* < 0.001
		10^6^			Group	57	3		87.60 ± 2.13	
		10^7^			Group	59	3		81.22 ± 4.74	
Manduca sexta L. (Sphingidae)	2	0	diet incorporation	1,000*	Group	90	4	17	4.78 ± 2.31	χ^2^ = 191.95, df = 1, *p* < 0.001
		10^3^			Group	56	3		19.23 ± 15.54	
		10^4^			Group	58	3		20.87 ± 5.43	
		10^5^			Group	72	4		6.29 ± 2.66	
		10^6^			Group	78	4		12.13 ± 4.80	
		10^7^			Group	77	4		72.24 ± 14.23	
		3*10^7^			Group	78	4		100.00	
Loxstege sticticalis L. (Crambidae)	2	0	diet incorporation	1,000*	Group	47	3	10	5.73 ± 3.07	χ^2^ = 0.44, df = 1, *p* = 0.504
		10^6^			Group	48	3		17.37 ± 5.60	
		10^7^			Group	51	3		5.66 ± 3.20	
Galleria mellonella L. (Pyralidae)	4	0	diet incorporation	1,000*	Group	50	5	10	0.00	No effect
		10^7^				50	5		0.00	
Helicoverpa armigera Hubner (Noctuidae)	2 to 3	0	diet incorporation	5	Individual	21	1	11	28.6	No effect
		10^7^								
					Individual	30	1		10	
*Dendrolimus sibiricus* Tsch. (Lasiocampidae)	2	0	drop-feeding	0.5	Individual	30	1	12	6.67	χ^2^ = 51.95, df = 1, *p* < 0.001
		10^3^			Individual	30	1		26.66	
		10^4^			Individual	30	1		20.00	
		10^5^			Individual	30	1		36.66	
		10^6^			Individual	30	1		83.33	
		10^7^			Individual	30	1		73.33	

a *per larvae group.

DsCPV-1 manifested high effectiveness with the optical brightener. When the adjuvant was added to a DsCPV-1 suspension at a concentration of 0.5%, it reduced LD_50_ values for both tested host species, although for *L. dispar* this reduction was much more pronounced, and LD_50_ for mixtures with adjuvants were eventually comparable between the two host species (Fig. 3a). The addition of the brightener also positively affected the speed of killing of the tested hosts. This effect was more significant for the main host (the speed was reduced more than 2-fold) compared with the alternative host (Fig. 3b).

## DISCUSSION

Our results indicate that the genus CPV contains some species that hold extra promise for pest management owing to their high virulence to the main host, high productivity (data of this study) even though this virus replicates only in midgut tissue (9–11), and broad specificity to lepidopteran host species. The assessment of cumulative mortality of *D. sibiricus* larvae (main host) revealed that even at a dose as low as 0.5 polyhedra per larva (i.e., only half of the individuals received a viral inoculum), the virus can kill half of the treated population. This high level of infectivity could be a consequence of the high sensitivity of the main host or/and successful horizontal transmission of the virus between individuals owing to the presence of mature occlusion bodies in host feces, as we observed in this study and as reported earlier (23). The virulence of the new strain, DsCPV-1, is much higher than that of another closely related strain, CPV-1 (derived from other hosts [8, 24]), and higher than the virulence of the CPV genus in general (24–29).

**FIG 5:**
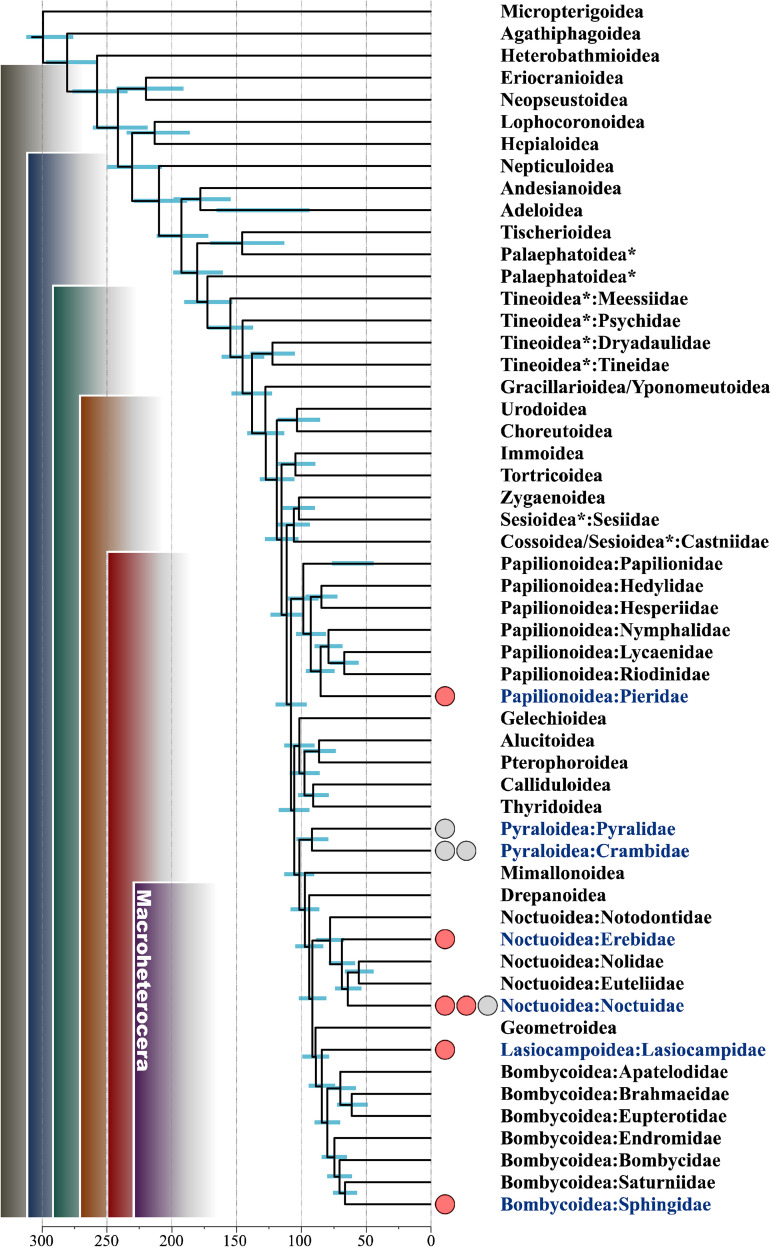
Host range of DsCPV-1 mapped onto the phylogenetic tree of Lepidoptera. The original maximum-likelihood tree were taken from Kawahara et al. (30), and collapsed up to the family/superfamily level. The status of the analyzed lepidopteran species is designated by red (susceptible species) and gray (resistant species) circles at the leaves of the corresponding family. Nonmonophyletic superfamilies are indicated by asterisks. Blue bars depict 95% credibility intervals of node ages.

One of the most important findings in this study is the unique feature of DsCPV1: an extra wide host range within the order. DsCPV-1 successfully infected six of 10 assayed lepidopteran species belonging to five families (Erebidae, Sphingidae, Pieridae, Noctuidae, and Lasiocampidae) by initiating normal pathogenesis in a susceptible host. We indicate the actual sensitive hosts within the lepidopteran evolutionary tree (Fig. 5), and most of them belong to different families within the Obtectomera clade, a relatively young clade within the order Lepidoptera (30). The Pyraloidea_clade shows a pattern of tolerance to the DsCPV-1 strain although a more thorough assessment using different dosages is needed to confirm this observation. Interesting results were obtained for members of the family Noctuidae, where different representatives displayed different levels of susceptibility. This phenomenon can be explained by the narrow window of opportunity (in terms of DsCPV-1 virulence) in the case of alternative hosts. For example, in the case of *M. sexta*, the full range of host response (i.e., from minimum to maximum mortality) was observed within 1 order of magnitude of viral titer dilution, while this window was much wider for the initial host (Table 1). Thus, the doses of DsCPV-1 higher than those used in the present study would cause mortality of *H. armigera*, but such high doses would not be operationally practical due to their high cost. A relatively broad host range has been documented for another CPV, Cypovirus 17, strain UsCPV-17, isolated from the mosquito *Uranotaenia sapphirina* (15). However, in that study, the different hosts tested belonged to a single family, while in our study, we identified susceptible hosts in different families within the order Lepidoptera. Thus, the scale of the host range of DsCPV is significantly higher than that previously found for other CPVs. The virulence of DsCPV1 toward alternative hosts was not as high as that toward the main host but is still comparable with the virulence of other entomopathogens used in pest management (13). It is known that representatives of the CPV-1 taxon have been isolated from different lepidopteran hosts, such as *D. punctatus* (31), Bombyx mori (32), and *L. dispar* (8); however, until our study, there has been no confirmation of such a wide host range for CPV-1 or even for the *Baculoviridae* family. Phylogenetic analysis of the sequenced DsCPV-1 genome showed that it most likely has arisen as a consequence of a recombination event between strains DpCPV1 and LdCPV-1, involving a replacement of the third segment of the DpCPV-1 genome with its LdCPV-1 homolog. It was recently reported that such evolutionary events as reassortment and intragenic recombination are widespread among RNA viruses, including CPVs, and serve as a mechanism of adaptation to changing environmental conditions, including host range expansion (33). The possibility of such an evolutionary mechanism is further supported by evidence that coinfection with several strains of CPVs occurs in natural populations (8, 34). Whether such an evolutionary scenario is responsible for the broadening of DsCPV-1’s host range and whether it involves the A-spike protein (responsible for the penetration function of the virus) in host recognition, remains to be determined.

This fundamental finding may form the basis for the following practical implications. First, the DsCPV-1 strain reported here may be considered an agent for population management of economically important lepidopterans pests with wide host susceptibility within the order. This means that a universal scheme of production of a viral insecticide may be implemented to create a viral product for a broad pest control market. This will positively affect the price of the insecticide and its profit margin. Second, it is possible to organize mass production of this strain *in vivo* in the most economically suitable inexpensive host species (the current problem with mass cultivation of entomopathogenic viruses in cell lines [the cost of the culture medium] is not solved yet). For example, in our study, *M. sexta* proved to be a better candidate for this purpose because it has large quickly developing larvae, does not have diapause, and can be reared on the relatively inexpensive standard diet. The passaging of DsCPV-1 via *M. sexta* (i.e., an alternative host) preserved the pathogenicity toward the main host. We did not quantitatively analyze the virulence against *D. sibiricus* after the passaging via *M. sexta* in this study because of the limited number of *D. sibiricus* larvae available for the study. Even though the passaging through an alternative host will decrease the virulence toward the main host or another susceptible lepidopteran species, the virulence will still be acceptable as long as the cost of virus propagation is attractive for its production. Thus, all the above findings about DsCPV-1 should considerably increase the flexibility of viral-insecticide production and are suggestive of good prospects of DsCPV-1 as a biological insecticide against lepidopteran pests with wide applicability because this virus has a true regulatory effect on pest population dynamics.

Our data are consistent with the results of another study (14), which indicates that adding optical brighteners to CPVs significantly (by several orders of magnitude) enhances a virus’s virulence toward the host and expands the host range (24, 35). This synergic effect of optical brighteners has also been documented for many baculovirus-lepidopteran host systems (36–38) and is explained by the temporary and reversible damage it causes to the peritrophic membrane of the insect gut, thereby increasing its penetrability by virions (36) and/or preventing sloughing of infected midgut epithelial cells (39). As applied to the present study, brightener may have increased the susceptibility of alternative hosts (that would otherwise display low sensitivity) up to the average susceptibility of the main host (which is normally very high). Thus, the use of an optical brightener(s) at low concentrations as an adjuvant for DsCPV-1 should widen the range of target hosts with reasonable virulence toward them. Moreover, the optical brightener here significantly decreased LT_50_ of the hosts, and this parameter is also important for the protective ability of pest control products. As mentioned in the Introduction, at present, there are microbial products on the pest control market that possess a similar set of features. These are dominant insecticides based on B. thuringiensis (1). According to general knowledge about CPV characteristics, i.e., good capacity for vertical transmission (40), fairly good resistance to environmental factors owing to the polyhedral crystal protein (41), relatively fast infectious-disease course, and a quick antifeedant effect on the host owing to gut damage (6, 41), our new highly virulent strain DsCPV-1 may be a viable competitor to B. thuringiensis products and could be more attractive than narrow-specificity Baculoviridae products. Moreover, owing to vertical transmission via surviving hosts, this strain may cause transgenerational downregulation of pest population size (which is impossible for Bt-based products). This ability is crucial for the control of polyvoltine insect pest species.

In light of DsCPV-1’s broad host range, an important question is the potential risk of its use for nontarget species, including humans. Even though some genera of the family Reoviridae can infect vertebrate or even mammalian organisms (10, 11), representatives of the Cypovirus genus have not ventured outside the class Insecta to find hosts (6, 9–11). Artificial inoculation of vertebrate cell lines by CPV does not lead to successful replication of the virus, indicating that mechanisms of vertebrate resistance against CPV are different from those associated with membrane penetration (9). We are currently assessing the infectivity of DsCPV-1 toward hydrobionts, pollinators, and entomophagous invertebrates, and our results thus far support the positive prospects regarding the biosafety of this virus (unpublished data). These results will be presented in a separate article.

In conclusion, we would like to draw virologists’ attention to CPVs in general and to the CPV-1 group in particular for research on the host range of these viruses (of course, our work was limited to local lepidopteran species), their safety for nontarget species (although all hosts of CPVs are still within the insect class) (9, 10), and molecular mechanisms of host–pathogen interaction. In this report, we provide substantial evidence that some representatives of CPVs are promising candidates for practical applications, which will help to create an environmentally friendly tool for pest management at a reasonable cost in the future.

## MATERIALS AND METHODS

### Experimental design.

Our experimental approach involved several steps: (i) isolate and describe the morphological and molecular features of the isolated virus; (ii) reinfect specimens of the initial host species to document the virus’ pathological features; (iii) study the potential host range of this new viral strain, focusing on species of economic importance; (iv) study the interaction between the virus and a nontoxic chemical adjuvant-enhancer when tested against the original host species and alternative host species; and (v) assess the possibility of successfully infecting the initial host species after passing the virus via alternative host species (in the context of mass production technology).

**(i) Virus isolation.** The virus was isolated from dead *D. sibiricus* larvae collected in 2020 in fir-cedar forests located at the foothills of the Eastern Sayan (55.059652°N, 96.050832°E). About 500 overwintered fourth/fifth-instar larvae were field-collected in the spring and brought to the laboratory. Then, during the rearing of postdiapause larvae, we monitored mortality and found that about 100 out of 500 were dead. The first identification of the virus was performed by microscopic analysis of a rough imprint of the content of dead larvae on a histological slide. For subsequent manipulations with the virus, 50 dead larvae with obvious viral infection symptoms (i.e., presence of a layer of polyhedra without additional microorganisms such as bacteria or fungi) were crushed in distilled water, and then polyhedral occlusion bodies were separated from rough debris by filtration through gauze, with subsequent centrifugation at 20,000 × *g* for 20 min. The obtained virus samples were then used for microscopy, genomic sequencing, and bioassays. Only dead larvae were used for polyhedra isolation.

**(ii) Light microscopy.** An aqueous suspension of polyhedral occlusion bodies was examined under a light microscope (Axioscope 40, Carl Zeiss, Germany) with oil immersion at 1,000× magnification. Nomarski contrast was employed for better visualization of the shape of the occlusion bodies.

**(iii) Electron microscopy.** The extracted occlusion bodies were fixed in suspension by the addition of an equal volume of an 8% paraformaldehyde solution. The mixture was incubated at 4°C for 24 h. Then the polyhedra were washed with Hanks’ solution, additionally fixed with a 1% osmic acid solution, dehydrated in increasing concentrations of ethanol and acetone, and embedded into the Epon–Araldite mixture. Semithin and ultrathin sections were obtained on a Reichert-Jung microtome (Austria). Semithin sections were stained with an azure-II solution, viewed under an AxioImager Z1 light microscope (ZEISS, Germany), and areas of interest were selected for subsequent ultrastructural analyses. Ultrathin sections were counterstained with uranyl acetate and lead citrate. Electron microscopy was carried out using a JEM 1400 electron microscope (Jeol, Japan). Photos were taken with a Veleta digital camera (SIS, Germany). Image analysis was performed in the iTEM software (SIS, Germany).

**(iv) Complete genome sequencing.** Nucleic acids were extracted from purified viral polyhedra using the ExtractRNA reagent (CJSC Eurogen, Russia) following the manufacturer's instructions. Two μL of aqueous glycogen (20 mg/mL) were used as a co-precipitator. The precipitate was dissolved in 30 μL of deionized water and utilized as a template in a reverse-transcription reaction to synthesize cDNA according to the SISPA protocol (Sequence-Independent, Single-Primer Amplification) and according to Djikeng et al. (42). Synthesized cDNA fragments were purified using AMPure beads (Beckman Coulter). The concentration of the DNA was determined with the Qubit dsDNA HS assay kit on a Qubit 3.0 fluorometer (Thermo Fisher Scientific). Next-generation sequencing libraries were prepared from the samples using the NEB Next Ultra II FS DNA Library Prep Kit for Illumina. Paired-end sequencing (2 × 250 bp) was performed on the MiSeq (Illumina) platform with the MiSeq reagent kit v2 (Illumina), resulting in 637- to 1,038-fold genome coverage.

Genome assembly was conducted in the MIRA (v.4.9.6) assembler with default parameters. Multiple-sequence alignment of CPV genomes was performed using the MAFFT (v7.407) algorithm. Maximum likelihood trees were inferred using RAxML-NG (43) within the GTR (G+I) model. Branch support was estimated by the bootstrap method (1,000 replications).

Prediction of recombination signals and evaluation of their statistical significance were conducted by the 3SEQ (44) algorithm.

NCBI GenBank accession numbers for the complete genomes of type 1 CPVs used in the study are provided in Table S1.

**(v) A bioassay of DsCPV-1 against the main host species: *D. sibiricus*.** *(a) 5.1. Half-lethal dose and median lethal time.* We used the population of *D. sibiricus* collected in fir-cedar forests located at the foothills of the Eastern Sayan. For the bioassay, we obtained the next generation (i.e., F1) of the insects under lab conditions. The larvae were reared at 24°C and 40% to 60% relative humidity under the 16:8 light:dark regime and were fed 2-year-old shoots of the fir *Abies sibirica* Ledeb.

For infection, we conducted a serial dilution experiment. In particular, the following virus concentrations in aqueous suspensions were employed: 10^7^, 10^6^, 10^5^, 10^4^, and 10^3^ polyhedra per milliliter. Polyhedra were isolated as described above. To inoculate the larvae with the virus, the droplet feeding method was applied (45). Second-instar larvae were individually fed with 0.5 μL of a suspension of the virus in a 10% aqueous sucrose solution. For better visualization, the drinking suspension was colored with a food dye. Larvae of the control group were fed a virus-free solution of sucrose. For each dose, we used 30 individuals. Mortality was recorded daily until pupation. The lethal doses required to kill 50% (LD_50_) of the inoculated larvae were determined using the *drc* software package (46). Differences in LD_50_ were considered statistically significant when fiducial limits did not overlap. Median lethal time (LT_50_) was determined by Kaplan–Meier survival analysis followed by the logrank test with Holm–Sidak adjustment.

*(b) 5.2. DsCPV-1 productivity.* For this assay, we used larvae collected in the larch forests of the Baikal region because the stock from the Sayan *D. sibiricus* population was limited in this study. The larvae were reared under the same conditions as described above (except for the host plant) and were fed the shoots of the larch *Larix sibirica* Ledeb.

For infection, fifth-instar larvae were individually fed 2 μL of a suspension of the virus in a colored 10% aqueous sucrose solution. The virus concentration in the suspension was 2 × 10^7^ polyhedra/mL. The infected and control groups contained 30 individuals each. The polyhedra were isolated from dead larvae by crushing a larvae body in a fixed volume of water. Then, the suspension of the homogenate (without filtration to avoid the loss of occlusion bodies) was diluted, and the occlusion bodies were counted on a hemocytometer at 400× magnification. Viral productivity was determined as the number of polyhedra per larva.

**(vi) Determination of the host species range.** The experiment involved larvae of nine species of Lepidoptera from six families (except for the main host). The choice of host species was based on two main principles: (i) the economic importance of the lepidopteran pest and (ii) the evolutionary distance between families (to estimate the coverage of susceptible host species). Peroral infection was carried out in two ways: drop-feeding and diet-incorporation. Drop-feeding consisted of feeding the insects a drop of a virus suspension (see Table 1 for each species) in a colored 10% sucrose solution. If the larvae refused the sweet droplet, then the infection was implemented by contamination of their diet, i.e., diet-incorporation. In the latter case, the scheme of infection for all species was as follows: 1 mL of the virus suspension was applied with a brush to feed intended for one group of insects. The amount of infected feed was such that a group of insects could eat it before it dried. After the larvae ate the contaminated feed or drank the droplet, they were placed in containers in accordance with the population density optimal for the corresponding species. Virus concentrations in suspensions during the infection of different species were uniform; however, with diet-incorporation, the concentration was higher than that employed for the drop-feeding in order to adjust the regimen for the losses seen in this method when the larvae did not finish their feed.

Species, larvae instar, concentration of the virus in a suspension, group assignment of insects, and the number of assay days are shown in Table 1.

*(a) Lymantria dispar.* *L. dispar* eggs were collected in the spring from wild birch stands in Novosibirsk Oblast, Russia. Next, the eggs were kept in a refrigerator at 4°C during winter diapause. The hatching of larvae was synchronized with the budburst of birch leaves (first third of May) according to Martemyanov et al. (47). After hatching, the insects were reared on cut branches of the silver birch Betula pendula Roth. The larvae were reared at 22°С, at natural humidity, and under the natural photoperiod.

*(b) Pieris brassicae.* Its eggs were collected in a cabbage field (one of the untreated fields) on a farm in Novosibirsk Oblast, Russia. The lower leaves of white cabbage (*Brassica oleracea* L.), grown in the laboratory, served as feed for the insects. Larvae were reared at 24°С to 25°С and 50% to 60% humidity under the natural photoperiod.

*(c) Ostrinia nubilalis.* We used insects from a lab population at the All-Russian Institute of Plant Protection (St. Petersburg, Russia). The rearing of larvae was performed on an artificial diet following the procedure of Frolov and coauthors (48).

*(d) Hyphantria cunea.* Larvae of the fall webworm *H. cunea* were collected on mulberry trees in Krasnodar Oblast (Russia) in August 2021 at the egg stage. Larvae were maintained in plastic 2-L containers and fed with mulberry leaves at 24°C under the natural light/dark regime.

*(e) Mamestra brassicae.* In this experiment, larvae of the first (F1) generation of the laboratory population were used. The larvae of the parental population were collected in a cabbage field on a farms in Novosibirsk Oblast, Russia. Lower leaves of white cabbage (*B. oleracea* L.), grown in the laboratory, served as feed for the insects. The insects were reared at 24°С to 25°С and 50% to 60% humidity under the natural photoperiod.

*(f) Manduca sexta.* Insect eggs together with artificial feed and the protocol of rearing were kindly provided by T-REX Co. (Russia). The insects were reared at 24°С to 25°С and 50% to 60% relative humidity under the 16:8 light:dark regime.

*(g) Loxostege sticticalis.* Larvae of the second (F2) generation of the laboratory population were used. The larvae of the parental population were collected on a forest edge in Krasnoyarsk Krai (Russia). The larvae were reared at 24°C and a relative humidity of 40% to 60% under the 16 h:8 h light:dark regime and were fed burdock *Arctium lappa* L. leaves.

*(h)* Galleria mellonella. Insect larvae were kindly provided by the insectarium of the Institute of Systematics and Ecology of Animals, the Siberian Branch of the Russian Academy of Sciences (Novosibirsk). Larvae were reared at 28°C and 60% relative humidity on a 12 h:12 h light:dark cycle and were fed an artificial nutrient medium. A detailed description is given by Dubovskiy et al. (49).

*(i)* Helicoverpa armigera. Larvae of the first (F1) generation of the laboratory population were used. Pupae of the parental population were collected in Uzbekistan. The larvae were reared on an artificial diet under natural illumination conditions.

**(vii) Challenging the main host *D. sibiricus* by the virus passaged through the *M. sexta* host.** *(a) 7.1. Passaging and virus isolation from M. sexta.* Middle-instar larvae of *M. sexta* we infected by the initial virus via diet-incorporation. The feed was supplemented at the concentration of 10^7^ polyhedra/mL, 1 mL per 20 larvae. Dead larvae were harvested and crushed, and the virus was purified according to the procedure described in subsection (i) Virus isolation.

*(b) Challenging the main host D. sibiricus by the virus passaged through M. sexta.* Second-instar larvae of *D. sibiricus* were employed for this purpose. We used larvae of a filial generation hatched in the laboratory from the parental population that was collected in fir-cedar forests located at the foothills of the Eastern Sayan. The larvae were reared at 24°C and 40% to 60% relative humidity under the 16 h:8 h light:dark regime and were fed 2-year-old shoots of the fir *A. sibirica*.

For infection, second-instar larvae were individually fed 10^4^ polyhedra per larva following the protocol described above. Larvae of the control group consumed a virus-free solution of sucrose. The experiment involved 14 experimental and 14 control individuals. We could not use more larvae for this bioassay because of the limited number of insects. The highest dose was chosen to qualitatively confirm the effectiveness of the virus against the main host. Mortality was recorded daily until the last individual died.

**(viii) Increasing the effectiveness of the virus by optical brightener Blankophor.** Larvae of three lepidopteran species were subjected to this experiment: *D. sibiricus*, *L. dispar*. Drop-feeding was utilized as the infection method to assess the effect of Blankophor (Miles Inc. Pittsburgh, PA, USA) on larval mortality from the viral infection, we conducted experiments with the following groups of larvae: (i) a 10% sucrose solution; (ii) a solution containing 0.5% of Blankophor and 10% of sucrose; (iii) a series of 10-fold dilutions of virus suspensions from 10^3^ to 10^7^ polyhedra/(mL of the 10% sucrose solution); and (iv) virus suspensions in a solution containing 0.5% of Blankophor and 10% of sucrose. The data are presented together with data on infection of the same species without the optical brightener.
