# Supplementary material for: A New Cypovirus-1 Strain as a Promising Agent for Lepidopteran Pest Control
Source: Microbiol Spectr. 2023 May 8;11(3):e03855-22. doi: 10.1128/spectrum.03855-22 (PMC10269911; doi:10.1128/spectrum.03855-22)
Supplement: Supplemental file 2 — Fig. S1 and S2. Download spectrum.03855-22-s0002.pdf, PDF file, 0.6 MB [file spectrum.03855-22-s0002.pdf]

Supplementary caption

Figure S1. Mortality dynamics of *D. sibiricus* larvae after infection with DsCPV-1

Figure S2. Unrooted phylogenetic trees inferred for segmented CPV genomes homologous to DsCPV1 segments (S1-S10), using maximum likelihood approach (RAxML-NG). Obvious topological incongruences between the inferred trees may be indicative of several reassortment events within the group. Amino acid sequences that correspond to entire or partial sets of RNA segments of CPVs or related reoviruses were used (GenBank IDs are contained in the tip labels). Some smaller segments of several viral genomes had no significant homology to other genomes and were excluded. Node labels are Felsenstein's bootstrap support values (1000 replicates). Host species abbreviations are as follows: Aa - *Antheraea assamensis*; Am - *Antheraea mylitta*; Br - *Biston robustus*; Bm - *Bombyx mori*; Co - *Choristoneura occidentalis*; Cb - *Clanis bilineata*; Cer - *Culex erythrothorax*; Dn - *Daphnis nerii*; Dp - *Dendrolimus punctatus*; Ds - *D. sibiricus*; Dm - *Drosophila melanogaster*; Ee - *Erinnyis ello*; Har - *Heliothis armigera*; Ii - *Inachis io*; Ll - *Lutzomyia longipalpis*; Ld - *Lymantria dispar*; Ob - *Operophtera brumata*; Op - *Orgyia pseudotsugata*; Sd - *Scaptodrosophila deflexa*; Tp - *Thaumetopoea pityocampa*; Tar - *Thyrinteina arnobia*; Tn - *Trichoplusia ni*; Lep - Lepidoptera; M - Mosquito; MAG - Metagenome Assembled Genome. RV - reovirus.

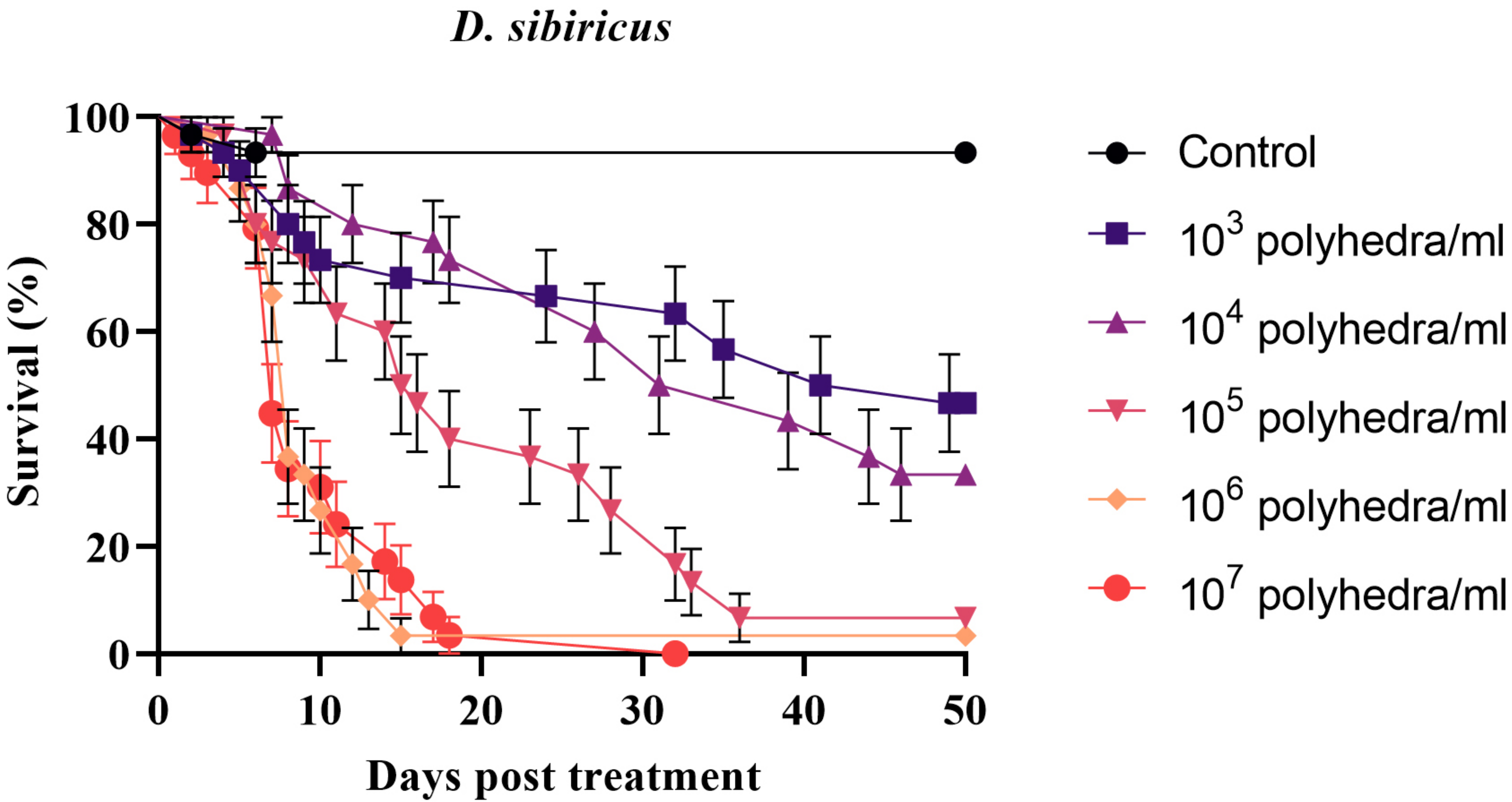

D. sibiricus
Survival (%)
100
80
60
40
20
0
0
10
20
30
40
50
Days post treatment
Control
10^3 polyhedra/ml
10^4 polyhedra/ml
10^5 polyhedra/ml
10^6 polyhedra/ml
10^7 polyhedra/ml

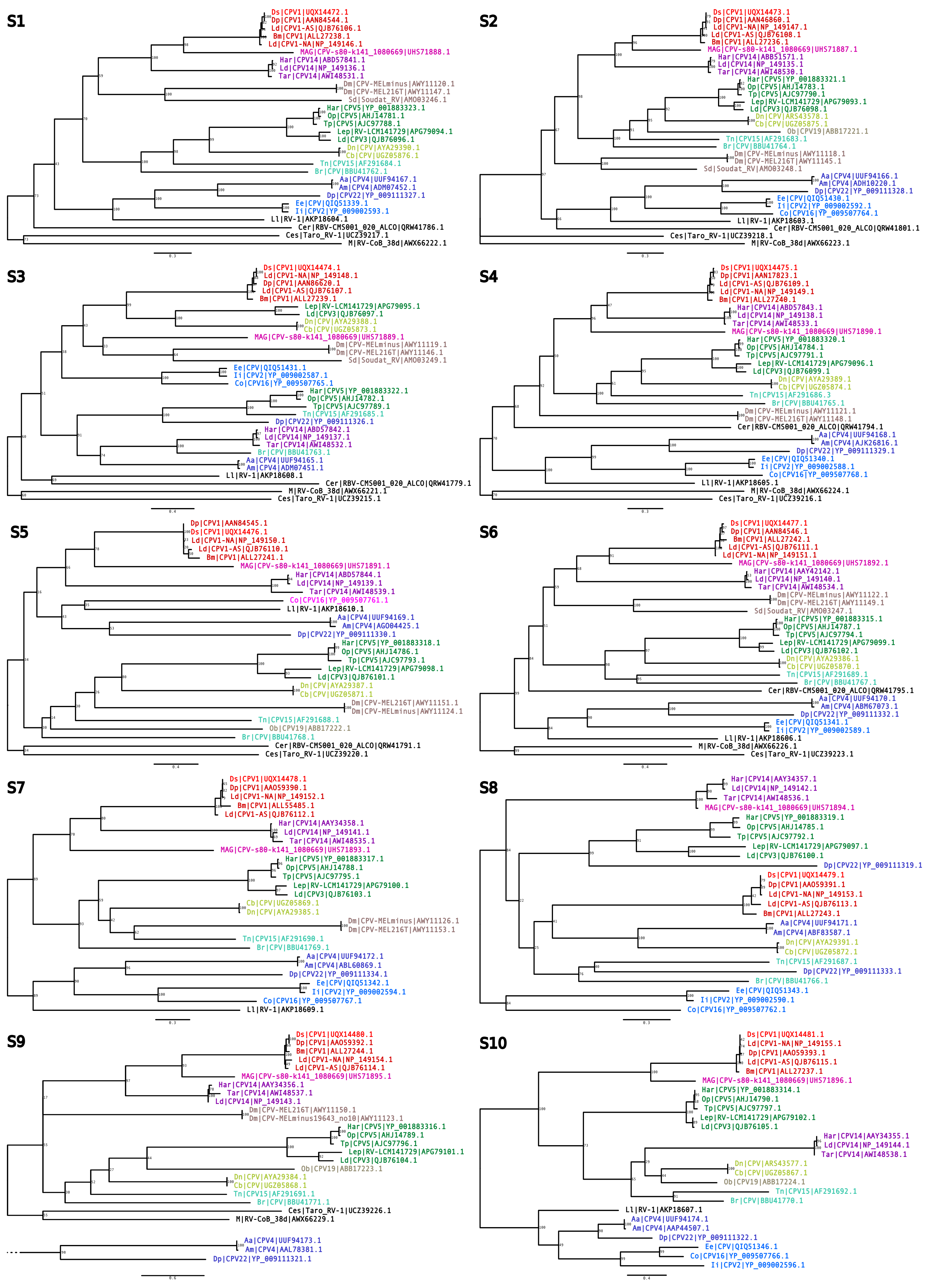
S1
Ds|CPV1|UQX14472.1
Dp|CPV1|AAN84544.1
Ld|CPV1-AS|QJB76106.1
Bm|CPV1|ALL27238.1
Ld|CPV1-NA|NP_149146.1
MAG|CPV-s80-k141_1080669|UHS71888.1
Har|CPV14|ABD57841.1
Ld|CPV14|NP_149136.1
Tar|CPV14|AWI48531.1
Dm|CPV-MELminus|AWY11120.1
Dm|CPV-MEL216T|AWY11147.1
Sd|Soudat_RV|AM003246.1
Har|CPV5|YP_001883323.1
Op|CPV5|AHJ14781.1
Tp|CPV5|AJC97788.1
Lep|RV-LCM141729|APG79094.1
Ld|CPV3|QJB76096.1
Dn|CPV|AYA29390.1
Cb|CPV|UGZ05876.1
Tn|CPV15|AF291684.1
Br|CPV|BBU41762.1
Aa|CPV4|UUF94167.1
Am|CPV4|ADM07452.1
Dp|CPV22|YP_009111327.1
Ee|CPV|QIQ51339.1
Ii|CPV2|YP_009002593.1
Ll|RV-1|AKP18604.1
Cer|RBV-CMS001_020_ALCO|QRW41786.1
Ces|Taro_RV-1|UCZ39217.1
M|RV-CoB_38d|AWX66222.1
S2
Ds|CPV1|UQX14473.1
Dp|CPV1|AAN46860.1
Ld|CPV1-NA|NP_149147.1
Ld|CPV1-AS|QJB76108.1
Bm|CPV1|ALL27236.1
MAG|CPV-s80-k141_1080669|UHS71887.1
Har|CPV14|ABB51571.1
Ld|CPV14|NP_149135.1
Tar|CPV14|AWI48530.1
Har|CPV5|YP_001883321.1
Op|CPV5|AHJ14783.1
Tp|CPV5|AJC97790.1
Lep|RV-LCM141729|APG79093.1
Ld|CPV3|QJB76098.1
Dn|CPV|ARS43578.1
Cb|CPV|UGZ05875.1
Ob|CPV19|ABB17221.1
Tn|CPV15|AF291683.1
Br|CPV|BBU41764.1
Dm|CPV-MELminus|AWY11118.1
Dm|CPV-MEL216T|AWY11145.1
Sd|Soudat_RV|AM003248.1
Aa|CPV4|UUF94166.1
Am|CPV4|ADH10220.1
Dp|CPV22|YP_009111328.1
Ee|CPV|QIQ51430.1
Ii|CPV2|YP_009002592.1
Co|CPV16|YP_009507764.1
Ll|RV-1|AKP18603.1
Cer|RBV-CMS001_020_ALCO|QRW41801.1
Ces|Taro_RV-1|UCZ39218.1
M|RV-CoB_38d|AWX66223.1
S3
Ds|CPV1|UQX14474.1
Ld|CPV1-NA|NP_149148.1
Dp|CPV1|AAN86620.1
Ld|CPV1-AS|QJB76107.1
Bm|CPV1|ALL27239.1
Lep|RV-LCM141729|APG79095.1
Ld|CPV3|QJB76097.1
Dn|CPV|AYA29388.1
Cb|CPV|UGZ05873.1
MAG|CPV-s80-k141_1080669|UHS71889.1
Dm|CPV-MELminus|AWY11119.1
Dm|CPV-MEL216T|AWY11146.1
Sd|Soudat_RV|AM003249.1
Ee|CPV|QIQ51431.1
Ii|CPV2|YP_009002587.1
Co|CPV16|YP_009507765.1
Har|CPV5|YP_001883322.1
Op|CPV5|AHJ14782.1
Tp|CPV5|AJC97789.1
Tn|CPV15|AF291685.1
Dp|CPV22|YP_009111326.1
Har|CPV14|ABD57842.1
Ld|CPV14|NP_149137.1
Tar|CPV14|AWI48532.1
Br|CPV|BBU41763.1
Aa|CPV4|UUF94165.1
Am|CPV4|ADM07451.1
Ll|RV-1|AKP18608.1
Cer|RBV-CMS001_020_ALCO|QRW41779.1
M|RV-CoB_38d|AWX66221.1
Ces|Taro_RV-1|UCZ39215.1
S4
Ds|CPV1|UQX14475.1
Dp|CPV1|AAN17823.1
Ld|CPV1-AS|QJB76109.1
Ld|CPV1-NA|NP_149149.1
Bm|CPV1|ALL27240.1
Har|CPV14|ABD57843.1
Ld|CPV14|NP_149138.1
Tar|CPV14|AWI48533.1
MAG|CPV-s80-k141_1080669|UHS71890.1
Har|CPV5|YP_001883320.1
Op|CPV5|AHJ14784.1
Tp|CPV5|AJC97791.1
Lep|RV-LCM141729|APG79096.1
Ld|CPV3|QJB76099.1
Dn|CPV|AYA29389.1
Cb|CPV|UGZ05874.1
Tn|CPV15|AF291686.3
Br|CPV|BBU41765.1
Dm|CPV-MELminus|AWY11121.1
Dm|CPV-MEL216T|AWY11148.1
Cer|RBV-CMS001_020_ALCO|QRW41794.1
Aa|CPV4|UUF94168.1
Am|CPV4|AJK26816.1
Dp|CPV22|YP_009111329.1
Ee|CPV|QIQ51340.1
Ii|CPV2|YP_009002588.1
Co|CPV16|YP_009507768.1
Ll|RV-1|AKP18605.1
M|RV-CoB_38d|AWX66224.1
Ces|Taro_RV-1|UCZ39216.1
S5
Dp|CPV1|AAN84545.1
Ds|CPV1|UQX14476.1
Ld|CPV1-NA|NP_149150.1
Ld|CPV1-AS|QJB76110.1
Bm|CPV1|ALL27241.1
MAG|CPV-s80-k141_1080669|UHS71891.1
Har|CPV14|ABD57844.1
Ld|CPV14|NP_149139.1
Tar|CPV14|AWI48539.1
Co|CPV16|YP_009507761.1
Ll|RV-1|AKP18610.1
Aa|CPV4|UUF94169.1
Am|CPV4|AGO04425.1
Dp|CPV22|YP_009111330.1
Har|CPV5|YP_001883318.1
Op|CPV5|AHJ14786.1
Tp|CPV5|AJC97793.1
Lep|RV-LCM141729|APG79098.1
Ld|CPV3|QJB76101.1
Dn|CPV|AYA29387.1
Cb|CPV|UGZ05871.1
Dm|CPV-MEL216T|AWY11151.1
Dm|CPV-MELminus|AWY11124.1
Tn|CPV15|AF291688.1
Ob|CPV19|ABB17222.1
Br|CPV|BBU41768.1
Cer|RBV-CMS001_020_ALCO|QRW41791.1
Ces|Taro_RV-1|UCZ39220.1
S6
Ds|CPV1|UQX14477.1
Dp|CPV1|AAN84546.1
Bm|CPV1|ALL27242.1
Ld|CPV1-AS|QJB76111.1
Ld|CPV1-NA|NP_149151.1
MAG|CPV-s80-k141_1080669|UHS71892.1
Har|CPV14|AAY42142.1
Ld|CPV14|NP_149140.1
Tar|CPV14|AWI48534.1
Dm|CPV-MELminus|AWY11122.1
Dm|CPV-MEL216T|AWY11149.1
Sd|Soudat_RV|AM003247.1
Har|CPV5|YP_001883315.1
Op|CPV5|AHJ14787.1
Tp|CPV5|AJC97794.1
Lep|RV-LCM141729|APG79099.1
Ld|CPV3|QJB76102.1
Dn|CPV|AYA29386.1
Cb|CPV|UGZ05870.1
Tn|CPV15|AF291689.1
Br|CPV|BBU41767.1
Cer|RBV-CMS001_020_ALCO|QRW41795.1
Aa|CPV4|UUF94170.1
Am|CPV4|ABM67073.1
Dp|CPV22|YP_009111332.1
Ee|CPV|QIQ51341.1
Ii|CPV2|YP_009002589.1
Ll|RV-1|AKP18606.1
M|RV-CoB_38d|AWX66226.1
Ces|Taro_RV-1|UCZ39223.1
S7
Ds|CPV1|UQX14478.1
Dp|CPV1|AAO59390.1
Ld|CPV1-NA|NP_149152.1
Bm|CPV1|ALL55485.1
Ld|CPV1-AS|QJB76112.1
Har|CPV14|AAY34358.1
Ld|CPV14|NP_149141.1
Tar|CPV14|AWI48535.1
MAG|CPV-s80-k141_1080669|UHS71893.1
Har|CPV5|YP_001883317.1
Op|CPV5|AHJ14788.1
Tp|CPV5|AJC97795.1
Lep|RV-LCM141729|APG79100.1
Ld|CPV3|QJB76103.1
Cb|CPV|UGZ05869.1
Dn|CPV|AYA29385.1
Dm|CPV-MELminus|AWY11126.1
Dm|CPV-MEL216T|AWY11153.1
Tn|CPV15|AF291690.1
Br|CPV|BBU41769.1
Aa|CPV4|UUF94172.1
Am|CPV4|ABL60869.1
Dp|CPV22|YP_009111334.1
Ee|CPV|QIQ51342.1
Ii|CPV2|YP_009002594.1
Co|CPV16|YP_009507767.1
Ll|RV-1|AKP18609.1
S8
Har|CPV14|AAY34357.1
Ld|CPV14|NP_149142.1
Tar|CPV14|AWI48536.1
MAG|CPV-s80-k141_1080669|UHS71894.1
Har|CPV5|YP_001883319.1
Op|CPV5|AHJ14785.1
Tp|CPV5|AJC97792.1
Lep|RV-LCM141729|APG79097.1
Ld|CPV3|QJB76100.1
Dp|CPV22|YP_009111319.1
Ds|CPV1|UQX14479.1
Dp|CPV1|AAO59391.1
Ld|CPV1-NA|NP_149153.1
Ld|CPV1-AS|QJB76113.1
Bm|CPV1|ALL27243.1
Aa|CPV4|UUF94171.1
Am|CPV4|ABF83587.1
Dn|CPV|AYA29391.1
Cb|CPV|UGZ05872.1
Tn|CPV15|AF291687.1
Dp|CPV22|YP_009111333.1
Br|CPV|BBU41766.1
Ee|CPV|QIQ51343.1
Ii|CPV2|YP_009002590.1
Co|CPV16|YP_009507762.1
S9
Ds|CPV1|UQX14480.1
Dp|CPV1|AAO59392.1
Bm|CPV1|ALL27244.1
Ld|CPV1-NA|NP_149154.1
Ld|CPV1-AS|QJB76114.1
MAG|CPV-s80-k141_1080669|UHS71895.1
Har|CPV14|AAY34356.1
Tar|CPV14|AWI48537.1
Ld|CPV14|NP_149143.1
Dm|CPV-MEL216T|AWY11150.1
Dm|CPV-MELminus19643_no10|AWY11123.1
Har|CPV5|YP_001883316.1
Op|CPV5|AHJ14789.1
Tp|CPV5|AJC97796.1
Lep|RV-LCM141729|APG79101.1
Ld|CPV3|QJB76104.1
Ob|CPV19|ABB17223.1
Dn|CPV|AYA29384.1
Cb|CPV|UGZ05868.1
Tn|CPV15|AF291691.1
Br|CPV|BBU41771.1
Ces|Taro_RV-1|UCZ39226.1
M|RV-CoB_38d|AWX66229.1
Aa|CPV4|UUF94173.1
Am|CPV4|AAL78381.1
Dp|CPV22|YP_009111321.1
S10
Ds|CPV1|UQX14481.1
Ld|CPV1-NA|NP_149155.1
Dp|CPV1|AAO59393.1
Ld|CPV1-AS|QJB76115.1
Bm|CPV1|ALL27237.1
MAG|CPV-s80-k141_1080669|UHS71896.1
Har|CPV5|YP_001883314.1
Op|CPV5|AHJ14790.1
Tp|CPV5|AJC97797.1
Lep|RV-LCM141729|APG79102.1
Ld|CPV3|QJB76105.1
Har|CPV14|AAY34355.1
Ld|CPV14|NP_149144.1
Tar|CPV14|AWI48538.1
Dn|CPV|ARS43577.1
Cb|CPV|UGZ05867.1
Ob|CPV19|ABB17224.1
Tn|CPV15|AF291692.1
Br|CPV|BBU41770.1
Ll|RV-1|AKP18607.1
Aa|CPV4|UUF94174.1
Am|CPV4|AAP44507.1
Dp|CPV22|YP_009111322.1
Ee|CPV|QIQ51346.1
Co|CPV16|YP_009507766.1
Ii|CPV2|YP_009002596.1

Figure S1
